# Microeconomic Losses Due to Intimate Partner Violence Against Women (IPVAW): Three Scenarios Based on Accounting Methodology Approach

**DOI:** 10.3390/bs15070914

**Published:** 2025-07-04

**Authors:** Elena Mañas-Alcón, María-Teresa Gallo-Rivera, Luis-Felipe Rivera-Galicia, Óscar Montes-Pineda

**Affiliations:** Institute of Economic and Social Analysis, Faculty of Economics, Business and Tourism, University of Alcalá, 28802 Alcalá de Henares, Spain; maria.gallo@uah.es (M.-T.G.-R.); luisf.rivera@uah.es (L.-F.R.-G.); oscar.montes@uah.es (Ó.M.-P.)

**Keywords:** intimate partner violence against women (IPVAW), economic impacts, Spain

## Abstract

This article thoroughly examines the multidimensional consequences of intimate partner violence against women (IPVAW) and estimates the monetary costs associated with this kind of violence in Spain for 2022. Based on the accounting model approach, three alternative scenarios are proposed to quantify the direct tangible costs of IPVAW from a microeconomic perspective. Each scenario considers the out-of-pocket expenditures and the opportunity cost of lost income due to IPVAW, borne by the survivor women, their families and relatives, the public sector, and the private organizations. The study utilizes microdata from the latest Spanish Macro-survey on Violence Against Women, conducted in 2019 by the Government Office against Gender-Based Violence (Spanish Government). Results show the costs ranging from EUR 1.38 billion (the most conservative estimate) to EUR 3.01 billion (the highest estimate). Further research is needed to deepen understanding of the mechanisms by which violence affects the various domains and agents of society.

## 1. Introduction

Violence against women is a major public health problem and a violation of women’s human rights that can negatively affect women’s physical, mental, sexual, and reproductive health, both in the short, medium, and long term (WHO, 2021a). Still, the impacts of this violence do not end at the health level, also affecting the general well-being of women victims, offspring, and families and causing serious social and economic consequences for countries and societies (WHO, 2021b).

Intimate partner violence against women (hereinafter IPVAW) refers to any behavior by a current or former male intimate partner within the context of marriage, cohabitation, or any other formal or informal union that causes physical, sexual, or psychological harm (WHO, 2021b, p. 4). Although intimate partner violence can also be perpetrated against men, it is mainly perpetrated by men against women (WHO, 2021b, p. 4; EIGE, 2021, p. 5). Intimate partner violence is the most common form of gender-based violence experienced by women (WHO, 2021a).

IPVAW includes acts of physical aggression, such as slapping, hitting, kicking, and beating; acts of sexual aggression, such as forced intercourse and other forms of sexual coercion; psychological violence/abuse, such as intimidation, constant belittling, and humiliating; and other controlling behaviors, such as isolating a person from their family and/or friends, monitoring their movements, restricting their access to information and services, and not allowing them to work outside of the home (WHO, 2021b, p. 4).

Thus, IPVAW is not limited to physical and/or sexual aggression, as it is produced through a varied typology of violent episodes that also include those related to so-called psychological violence, which can be emotional, controlling, or economic. If these other types of violence are not taken into account, the broader part of the problem is not being considered.

Globally, the latest WHO estimates show that among ever-married/partnered women aged 15–49 years, 27% report having been subjected to physical and/or sexual violence by their partner at some point in their lives. Although IPVAW is more prevalent in the least developed countries (37% on average), high-income countries and areas also have very high prevalences (22% on average; 25% in Northern America, 23% in Northern Europe, and 21% in Western Europe) (WHO, 2021b, pp. 25–26).

In Spain, according to the most recent information available (DGVG, 2020; Mañas-Alcón et al., 2024), 14.2% of women of 16 years or older reported having been subjected to physical and/or sexual violence by their current or former partner at some point in their lives. The prevalence rises to 32.4% considering all the types of IPVAW (physical, sexual, and/or psychological).

IPVAW is, therefore, a problem that remains pervasive and persistent in the 21st century, and Spain is not an exception. In addition to the high and wide prevalence, cost studies estimate that the economic losses of IPVAW are substantial. Duvvury et al. (2004, 2013) and EIGE (2014, 2021) compiled a comprehensive range of these studies. For both reasons, studies evaluating the costs of IPVAW are increasingly valued internationally, and since the mid-1990s, they have received growing academic interest. They help to highlight the relevance of such violence, the significant losses it entails, and what society stands to gain if it were to be eradicated or at least significantly reduced.

### 1.1. Background Literature on the Impacts of IPVAW

Nowadays, an increasing number of studies are available in a wide range of countries and geographical areas, providing evidence of the significant adverse impacts and costs of IPVAW (or a closely related concept). An extensive collection of these studies since 1995 can be found at EIGE (2014, 2021) and Council of Europe (2012/2014), covering the Netherlands, Switzerland, Finland, the USA, Australia, England and Wales, Sweden, former Yugoslav, the Republic of Macedonia, Uganda, Marocco, France, Norway, Denmark, Bangladesh, Vietnam, Canada, Switzerland, Italy, Ireland, the United Kingdom, Spain, Germany, and UE-25, UE-27, and UE-28.

NCIP&C (2003), Walby (2004), Access Economics (2004), Duvvury et al. (2004), Envall and Eriksson (2006), and ICRW (2009) are examples of pioneering research. However, there are many recent studies (EIGE, 2021; Commonwealth Secretariat, 2019, 2020, 2022; European Parliamentary Research Services, 2021; Walby et al., 2020; Mañas-Alcón et al., 2019, 2024; Néréa et al., 2018; Ashe et al., 2017; Ornstein, 2017; Cavalin et al., 2015; Walby & Olive, 2014; Duvvury et al., 2013, among others).

The impacts of IPVAW are highly complex and multifaceted, involving many actors. This kind of violence not only entails pain and suffering but also very significant economic and social losses. Most studies tend to categorize these impacts into several spheres or domains, including health, legal, work and productivity, social welfare, and emotional, among others.

Focusing on health, IPVAW is considered a major public health issue with significant impacts on women and healthcare systems. Its effects extend beyond the health of female victims, also affecting the physical and mental well-being of their children who have experienced this traumatic situation (WHO, 1996).

Many studies focus on better understanding and estimating the health consequences for women in such violent circumstances (García-Moreno et al., 2005; WHO, 2013; Walby & Olive, 2014; Mañas-Alcón et al., 2019, 2024, among others). IPVAW is therefore recognized as a determinant of poor health outcomes for women. The potential relationship between IPVAW and health damage highlights various pathways through which this violence contributes to morbidity and mortality, including both direct or visible injuries and indirect or invisible ones (Mañas-Alcón et al., 2019). The heightened relative risk of injury among women experiencing IPVAW has been addressed in numerous studies (Sheridan & Nash, 2007; Hegarty et al., 2008; McCauley et al., 1995; Rachana et al., 2002; García-Moreno et al., 2005; WHO, 2013). Other research focuses on sexual health, presenting evidence of a higher likelihood of sexually transmitted infections (Jewkes, 2010) and increased exposure to abortion and having a low-birth-weight baby, thereby worsening reproductive health (WHO, 2013).

Several studies have analyzed mental health impacts (Black & Breiding, 2008; Black, 2011; Howard et al., 2010). In situations of continuous violence, the existence of the “battered woman syndrome” has been suggested (Walker, 2009), as it causes significant psychological impacts on women, which makes their capacity to react to aggression increasingly weaker, making them passive and intensely fearful of their aggressor partner. The explanation for this is related to the three phases of the “cycle of domestic violence” (Walker, 2009): phase of accumulated tension, phase of acute abuse, and phase of calm and reconciliation.

The children of assaulted women also have health problems, derived both from the environment of violence in which they live (often witnessing or hearing it) and, on occasion, from also becoming direct victims of the same aggressor. These health impacts are complex and vary according to age (Martínez León, 2015).

At times, IPVAW results in fatal outcomes, including homicides and suicides. International evidence indicates that, on average, 38% of all female homicides are attributed to gender-based violence (WHO, 2013, p. 2; Stöckl et al., 2013). A strong association is found between mental health problems resulting from partner rape and suicidal behavior (García-Moreno et al., 2005).

Consequently, psychological symptoms and/or physical injuries associated with IPVAW often appear early on, leading to frequent healthcare visits by affected women, thereby placing a substantial strain on healthcare systems (García-Moreno et al., 2005; WHO, 2013; Heise, 1996; McCauley et al., 1995; Bradley et al., 2002; Richardson et al., 2002). However, in many cases, healthcare professionals fail to identify this violence as the underlying cause of the symptoms (García-Moreno et al., 2005; WHO, 2013; MSSSI, 2012, 2017).

From the legal point of view, violent behaviors by men against their partners or former partners constitute a violation of women’s fundamental rights and are considered a criminal offence. Therefore, these behaviors must be investigated by the police and analyzed by the courts to determine the extent and severity of the incidents and, if necessary, to impose penalties and measures on the perpetrators while also providing protection and support measures for the victims. Additionally, these behaviors often involve family-related processes such as separations, divorces, custody, and other matters within the scope of civil justice. Consequently, IPVAW has significant legal implications through the judicial and security system, affecting victims, the public sector, and society (Zhang et al., 2012; Camarasa i Casals, 2009).

Access to justice is crucial for women to escape violent relationships and obtain legal protection. However, detection of such violence remains low, as many incidents are not reported or witnessed by authorities and therefore are not reflected in official records or incorporated into the cycle of actions and procedures within this domain. Walby (2004) and Zhang et al. (2012) highlighted the substantial difference between the actual occurrences of gender violence and the figures recorded in administrative registers.

The impacts of this domain are often analyzed from several fronts, typically including legal advice, police intervention, judicial processes, and penitentiary measures (Walby & Olive, 2014, p. 53; Zhang et al., 2012; Mañas-Alcón et al., 2019, 2024). Studies on the cost of crime, such as those by Jaitman and Torre (2017) and Dubourg et al. (2005), are essential references for applying to IPVAW cases.

The scope of the effects of IPVAW in the productive and labor sphere is also very significant and multidimensional, with a substantial number of studies focusing on these effects (Vara-Horna, 2013, 2015a, 2015b, 2016; Vara-Horna et al., 2017; Walby & Olive, 2014; Duvvury et al., 2013; Access Economics, 2004; Walby, 2004; Zhang et al., 2012; Nectoux et al., 2010). The mediating variable for most of the effects of IPVAW in this area is the deterioration of the victim’s health or morbidity.

It affects female survivors but also other individuals and agents in their environment, especially their family members and relatives, their perpetrators themselves, their employers, the public sector, and the third sector. The economic costs are particularly significant for the women survivors, employers, and the public sector (Vara-Horna, 2013, 2015a, 2015b, 2016; Vara-Horna et al., 2017; Walby & Olive, 2014; Mañas-Alcón et al., 2019, 2024). These effects include reduced labor participation, absenteeism, presenteeism, job loss, and difficulties in social and labor reintegration (Duvvury et al., 2013).

The impact of IPVAW on employers is associated with absenteeism, loss of productivity, and personnel management (Bradley et al., 2002; Richardson et al., 2002). Presenteeism, where workers attend their jobs but have reduced performance due to stress or anxiety, is a growing problem (Vara-Horna et al., 2017, p. 24). Perpetrators may also experience absenteeism and presenteeism, which impact company productivity (Vara-Horna et al., 2017). Although traditionally considered a private issue, more and more companies are implementing policies to address gender violence in the workplace, given its adverse effect on organizational climate and corporate reputation (Vara-Horna, 2015c, p. 55).

Women in the informal economy face greater vulnerability, as they lack access to benefits and may be forced to continue working under precarious conditions (Zhang et al., 2012). Moreover, although less numerous, some studies consider unpaid household activities within the estimation of the costs of gender violence (Zhang et al., 2012; Stern et al., 2013; Envall & Eriksson, 2006; Piispa & Heiskanen, 2000; NCIP&C, 2003; Mañas-Alcón et al., 2019, 2024).

Additionally, the economic losses suffered by the victims are compounded by the costs derived from barriers to accessing employment and training, which are fundamental aspects of enhancing their employability, achieving job stability, and taking advantage of professional promotion opportunities (Zhang et al., 2012).

It is essential to emphasize that paid employment is the primary avenue to achieving economic autonomy and breaking the cycle of violence. The lack of income and its control by women contributes to the perpetuation of IPVAW over time, as it prevents women from having the independence, confidence, and economic and financial self-control that would enable them to escape the spirals of violence, which can occur even when there is no longer cohabitation or physical proximity (Mañas-Alcón & Gallo-Rivera, 2020).

IPVAW impacts extend beyond the health, legal, and labor spheres: for example, home removal, shelter services, and the variety of social services needed by women suffering IPVAW and in situations of economic vulnerability (Zhang et al., 2012). The women survivors, the public sector, and third-sector organizations bear these costs.

In summary, from an individual point of view, talking about IPVAW sometimes means talking about loss of life, but, with a very high probability, it also means talking about injuries and damage to physical, reproductive, and/or mental health, sometimes constraining daily life; about police actions and more or less complex legal and penitentiary processes; about difficulties in accessing, seeking, or keeping a job and in receiving an income; about absences and distractions in the workplace; about the barriers in accessing education and loss of training opportunities and improvement of human capital; of loss of productivity in the business and the domestic sphere; of temporary incapacity to care for children and dependent family members; of forced relocations and removals; of fear, isolation and uncertainty, and pain and suffering, among others.

As a result of all these multidimensional impacts, IPVAW also generates multiple costs that should be made visible in due course. Providing measures of them through the estimation of monetary amounts involved is a task of the utmost relevance.

### 1.2. IPVAW Costing Estimation Methodologies

Some meta-reviews of cost studies, such as that of Duvvury et al. (2013) and Ashe et al. (2017), have indicated that methods for estimating IPV economic costs are continually being refined. Thus, IPVAW cost studies have generated various methodologies that encompass both microeconomic and macroeconomic perspectives (Raghavendra et al., 2017) as well as both demand- and supply-side perspectives (KPMG, 2014, p. 10).

Ashe et al. (2017) identified nine main approaches that allow for the quantification of the impacts of violence against women and to identify characteristics of households experiencing IPVAW and its impacts. The accounting method involves aggregating costs across sectors utilizing prevalence rates, service utilization rates, and average unit costs within each sector. Econometric analysis, commonly logistic or probit regression, is used to examine the relationship between violence against women and many of its associated outcomes. Propensity score matching is a non-parametric technique that compares the mean outcomes of those who experience violence and those who have not experienced violence. Quality of life losses encompasses disability-adjusted life years (DALY) and years of life lost. Population attributable fractions determine the proportion of a disease that could have been avoided if the population had never been exposed to a risk factor. Willingness to pay establishes the amount communities are willing to pay to avoid the risk of violence. Benefit/cost ratios summarize the overall value-for-money of a project or proposal. Gender-responsive budgeting analyzes government budgets and the budget cycle to identify the gendered impacts of budgetary decisions. Lastly, economic multipliers focus on the losses to economic growth resulting from violence, taking into account the structural interlinkages of the macroeconomy.

The suitability of the methods used depends on the nature of the costs to be estimated and the information available to assess them. Nevertheless, the accounting approach is one of the most widely used, mainly due to its systematization and ability to provide aggregate estimates (Ashe et al., 2017). The other methodologies present complementary approaches.

The accounting method can be applied to a wide variety of costs and in combination with other methodologies, such as the economic multipliers method (as in Mañas-Alcón et al., 2024). This methodology also enables a detailed disaggregation of the costs derived from medical care, social, and legal services (for an aggregate cost of preventing and responding to violence) as well as related losses due to foregone income resulting from reduced productivity or mortality (establishing foregone income) (Duvvury et al., 2013). This focuses on establishing a unit cost either through a bottom-up (based on detailed costs for providing a service) or top-down proportional approach (derived from an annual budget on a specific service and the proportion which would correspond to IPV) and data on the prevalence of violence and others like the number of incidents experienced in a year, activity days lost per incident, and average wage, among others (Duvvury et al., 2013). For example, in the case of service expenditures due to violence, the estimation following a bottom-up approach starts with the unit cost of each service and multiplies it by its frequency of use by victims, then adds these results to obtain the total cost (Willman, 2009).

This methodology has numerous applications. For example, the International Centre for Research on Women (ICRW, 2009) uses it for Uganda, Morocco, and Bangladesh, including estimates of health, police, justice, and social services costs. NCRVAW&C (2009) for Australia focuses on the costs of pain, suffering, and premature mortality as well as health, production-related, consumption-related, administrative, second-generation, and transfer costs. Zhang et al. (2012) applied to Canada a very detailed set of costs concerning the criminal and civil justice system, health, productivity losses for employers and households (in terms of wages, household services, and others), other personal costs (like damage or destroyed property), pain and suffering, and loss of life, among others. Duvvury et al. (2012) used it in Vietnam to measure costs associated with medical, police, court, shelter, legal aid, foregone earnings, and productivity loss. There was also an application of UNFPA (2015) to Egypt, which included tangible costs such as health services, property replacement, legal and judicial proceedings, shelter, local community services, missed domestic work, and missed working and school days, among others. It is also noteworthy that the IPVAW cost estimation for the United Kingdom (EIGE, 2021), based on Heeks et al. (2018), updated figures on lost economic output, health services, the criminal and civil systems, social welfare, personal costs, specialist services, and physical and emotional impacts. In the case of Spain, there have been two applications of the accounting model (Mañas-Alcón et al., 2019, 2024), including healthcare, legal-police, and labor-productive costs, among others.

Despite its advantages, its data requirements are a big challenge. It requires accurate data on the prevalence of IPVAW, victims’ use of services, other impacts of the violence, and a wide variety of unit costs. Consequently, many countries have significant gaps in the required data (Duvvury et al., 2013). However, it is particularly appropriate in Spain, where specialized surveys (Macro-survey of Violence Against Women) are available, (DGVG, 2020).

### 1.3. The Current Research

Despite efforts to address the problem, Spain also has a high prevalence of IPVAW. However, at the national level, only very recently have IPVAW cost studies arisen (Mañas-Alcón et al., 2019, 2024). Based on the second one, this research aims to provide monetary estimates of a comprehensive range of tangible direct costs associated with IPVAW in Spain for 2022. The estimation strategy adopted is based on the available literature background on the adverse outcomes of IPVAW and the methodological approaches to their quantification, as summarized earlier.

The accounting model methodology is used to measure these costs and highlight their distribution among different stakeholders and domains. Three alternative scenarios are proposed based, essentially, on various groups of women according to the type of IPVAW suffered. The third scenario is the broadest one and includes not only physical and sexual violence but also emotional violence and fear of the intimate partner.

The contribution enhances the understanding of the multidimensional impacts of IPVAW, the scope of its economic and social losses, the breadth of affected agents, and the sensitivity of the resultant costs to psychological violence. The results allow us to formulate some policy recommendations.

## 2. Methodological Issues and Materials

The study proposes a strategy for estimating the tangible direct costs of IPVAW in Spain for 2022 based on an accounting methodology. Thus, it considers the costs incurred by actors involved in or closely associated with this type of violence, having a market value or equivalent. It is based on a microeconomic approach that provides an overall monetary measure of the losses implied by IPVAW for Spanish society as a whole in that year (in terms of higher expenditures incurred and lower income received) and, at the same time, details on how they are distributed among the main agents that bear them and on the specific sphere or area in which they have occurred.

According to EIGE (EIGE, 2023) and the 2019 Macro-survey on Violence against Women in Spain (hereafter 2019 Macro-survey), a woman is considered a victim of IPVAW if she has suffered, as perpetrated by a man who is or has been her partner, at least one of the following types of violence: physical, sexual, psychological-emotional, psychological-control, or economic and/or is or has been afraid of him.

### 2.1. Microeconomic Approach to IPVAW Costs: General Methodological Aspects

The application of the accounting method requires first determining the types of costs to be considered and simultaneously defining which agents are affected. Since the costs are numerous and heterogeneous, they are presented grouped by area. The bibliographical background, the Spanish legal framework^1^, and the availability of information are crucial elements in determining these aspects.

This study examines the direct, tangible costs associated with IPVAW, encompassing expenditures incurred and income lost. The former corresponds to those expenditures incurred by different economic agents in anticipation, consequence, and response to IPVAW, including the costs incurred by the public health system to provide medical and hospital care to victims, including pharmaceuticals, expenditures on prevention and social services, and spending on counseling and legal assistance services as well as those of the police, judicial, and penitentiary systems. The latter consider the opportunity costs that correspond to the income lost due to the days spent by women survivors (and their families and relatives) away from work to attend to legal and health issues as a result of the violence suffered, the loss of employment, the reduced participation of women in the labor market, or those derived from not being able to carry out daily activities generally as they would in the absence of violence; also considered are the costs of managing absences from work and substitutions in the organizations, among others.

A total of 30 types of costs were estimated, which were first partially aggregated into four broad categories called domains (health, legal-police, labor-productive, and others) for each of the affected agents (women survivors, family and relatives, employers, public sector, and third sector). Finally, they were aggregated globally as a total measure of the tangible direct costs of IPVAW in Spain in 2022. Thus, with “*i*” representing the type of partial cost and “*j*” denoting the agent to which it applies, the total aggregation for every kind of cost is obtained as the sum of that cost “*i*” across all agents “*j*” Equation (1). On the other hand, the total cost for each agent “*j*” is the result of summing the costs borne by that agent across all “*i*” Equation (2). Finally, the total cost for Spain is calculated by adding all the partial costs for all agents, i.e., “*i*” and “*j*”, Equation (3).

(1) $${TC}_{i}=\sum_{j}c_{ij}$$

(2) $${TC}_{j}=\sum_{i}c_{ij}$$

(3) $${TC}_{i,j}=\sum_{i,j}c_{ij}$$

**Figure 1:**
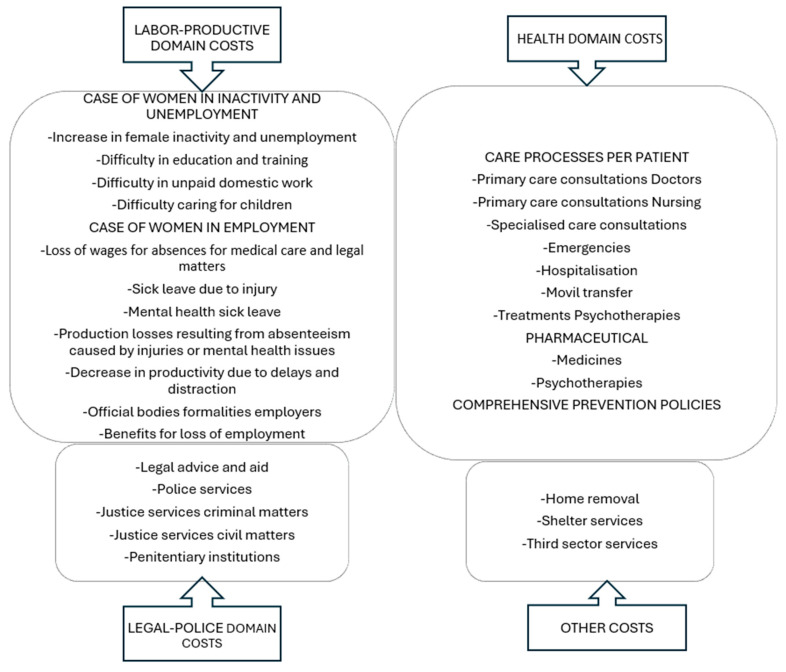
Details of the IPVAW economic costs by domain have been considered in the estimates. Source: The authors.

**Figure 2:**
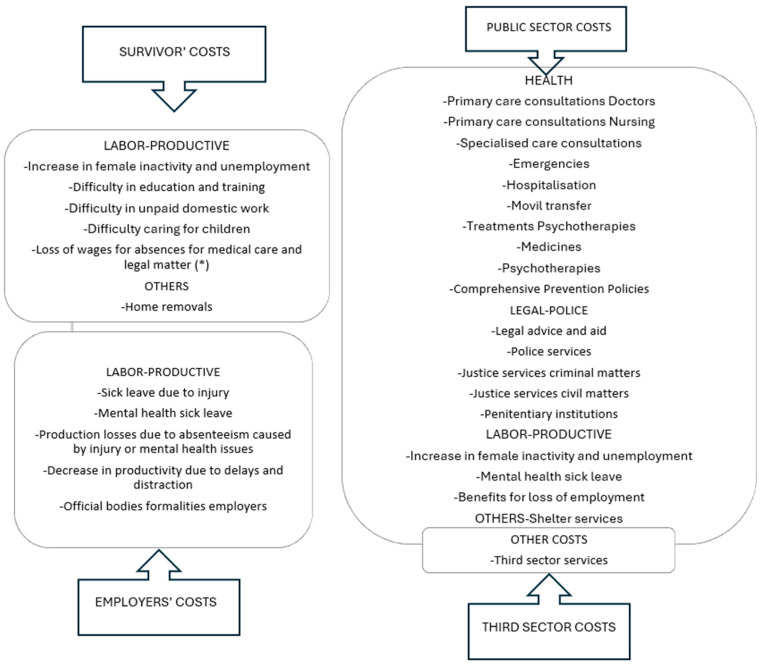
Details of IPVAW economic costs by affected agents that are considered in the estimates. * For this type of cost, those incurred by families and relatives are also estimated. Source: The authors.

Figure 1 and Figure 2 present the details of the costs, grouped by domain and by agent, respectively.

The reference period for estimating direct tangible costs is 2022. Its calculations are based on prevalence-year and unit cost figures defined for that year.

Information on the prevalence of IPVAW was obtained for most of the costs^2^ from the exploitation of microdata from the 2919 Macro-survey on Violence against Women 2019 (Macro-survey)^3^. The Government Office against Gender-Based Violence (DGVG in Spanish) conducted the Macro-survey. The microdata are publicly accessible, and the files are anonymized. The indicator that measures the cases of IPVAW incorporated in the estimates is the prevalence-year, which is defined as the number of women aged 16 years or older residing in Spain who, at least once during the 12 months before the interview, experienced any of the violent behaviors included in that form of violence. The factor to scale the sample prevalences offered by the 2019 Macro-survey to the population as a whole takes as a reference the total number of women aged 16 and over residing in Spain on 1 January 2022 (20,736,963, according to the Population Census), which allows us to approximate the population prevalence for the year 2022.

The population prevalences allow us to more deeply understand the different channels of transmission of the violent experience suffered by women themselves, their families and relatives, or their employers as well as the burden on public services (for example, different types of health care or social services) or civil society. To achieve this objective, the 2019 Macro-survey provides very relevant information on these effects in terms of injury, damage, or disability; absences from the workplace or place of study; recourse to medical or psychological services; and consumption of substances such as medication, alcohol, or drugs; among others. It was supplemented with other sources, as described in detail in Section 2.1.

The average cost was used to measure the unit cost for each type of impact. A bottom-up approach was employed, which begins with the identified unit cost and then scales it by multiplying it by the number of incidents. However, a top-down proportional approach was employed for the legal-police domain, which determines what part of the total public budget for these matters, in general, is a consequence of the recorded episodes of IPVAW. The Macro-survey 2019 does not provide information to establish unit costs, so we resorted to a wide range of official sources (INE, the Government Office against Gender Violence, the General Council of the Judiciary, the Ministry of the Interior, the Ministry of Health, and the Ministry of Labour and Social Economy, among others) or previous studies, almost always referring to Spain or one of its regions.

Finally, it is worth noting that this methodology was applied considering three possible scenarios. They differ mainly by the size of the groups of women victims on which they are built, according to the type and intensity of IPVAW suffered or the impact it causes on their health. Scenario A considers women who have suffered physical and/or sexual IPVAW (hereinafter PSV) with limiting injuries; scenario B includes women who have experienced PSV differentiated according to the severity of violence; scenario C considers women victims of physical, sexual, and emotional^4^ violence and/or those who are or have been afraid of an intimate partner (hereinafter PSEVF).

**Figure 3:**
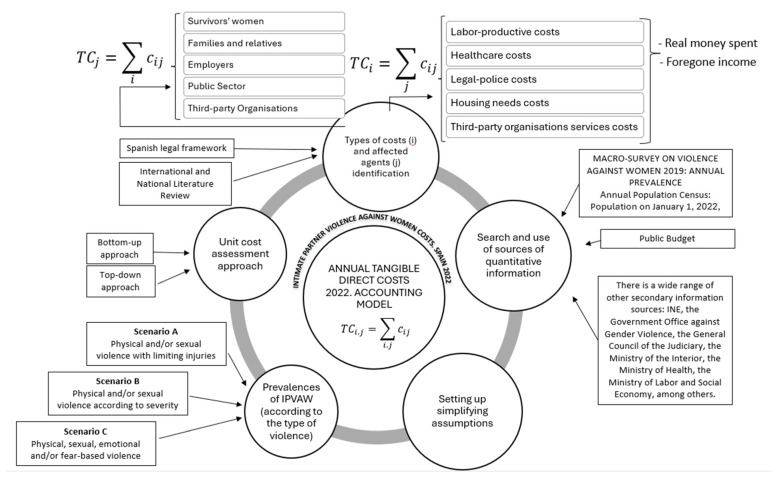
Direct tangible costs of the IPVAW estimation methodology scheme: Accounting model for Spain. Source: The authors.

Figure 3 summarizes the methodological aspects.

### 2.2. Specific Methodological Aspects in Each Domain

#### 2.2.1. Healthcare Domain

This domain assesses the impact of IPVAW on the health of women survivors, including their physical, sexual, reproductive, and mental health. It examines their subsequent use of public health services, including the frequency of their use.

For this purpose, estimates of the unit cost are calculated based on the Official State Bulletin, which serves as the reference source for drug prices and the prices and costs of other health services, such as diagnostic tests or rehabilitation services. Regarding the pharmaceutical costs associated with treatments for IPVAW victims, studies by Cruz Roja Española (2017), National Health System, Aguirre Martin Gil (2008), and Servicio Murciano de Salud Pública (2010) were utilized.

This unit cost is applied to the affected women as well as the frequency of use figures. The aggregation of all the amounts thus obtained provides an estimate of the differential costs of this violence on the public health system in 2022. The budget allocated by the state for the prevention of IPVAW was also taken into account. All the costs estimated in this pathway were assigned to a single agent, the public sector, as the amounts corresponding to the disbursement made by the victims were not included.

These costs are grouped under three main headings: (1) healthcare (including primary and specialized care); (2) pharmaceutical costs; and (3) costs for prevention policies.

Two alternative estimates were implemented for the first two types (1 and 2) based on the breadth of IPVAW types considered. The first applies unit costs to the number of women who have experienced PSV and have reported contacting health services (in the calculation of total costs, it is added to scenarios A and B described above). The second applies these unit costs to the number of women who have experienced PSEVF and have reported contacting health services (in the calculation of total costs, it is added to scenario C).

The total cost of healthcare and pharmaceuticals is the result of multiplying the unit cost of the service or medicine by the number of individuals requesting them and the frequency of use.

For health care costs, the determination of the number of cases of women victims demanding health services, both for physical, sexual, and reproductive health damage and mental health damage, was based on the 2019 Macro-survey. In the next step, the primary care or specialized care referrals were based on the Ministry of Health’s (Ministerio de Sanidad, 2023). Thirdly, an approach to the use of physician and nursing services (in the primary care setting) and outpatient consultations, emergencies, hospitalizations, and ambulances (in the specialized care setting) was applied using the study by Aguirre Martin Gil (2008) for the Community of Madrid. In addition, according to the literature, women who have suffered these forms of violence and have more significant damage to their mental health require specialized psychotherapy for a more extended period (Servicio Murciano de Salud Pública, 2010). To determine this demand, a more specific group of victims of these forms of violence was selected, who, in addition to contacting psychological/psychiatric services, gave an affirmative answer to a combination of symptoms of their affected mental health status in the 2019 Macro-survey.

Finally, costs associated with prevention activities were approximated at the state level by the budget allocated to the integrated actions for the prevention of gender-based violence of the Spanish Ministry of Health (Program 232C (Comprehensive Prevention) and Program 313B-481 (Women’s Health Observatory of the Ministry of Health) in 2022.

#### 2.2.2. Legal-Police Domain

This domain estimates the costs arising from the increased use of services provided by the public sector in the legal sphere in response to IPVAW detected in official records. These are tangible direct costs that fall on the public sector at its different territorial levels, classified into four categories: (1) free legal aid costs; (2) police costs; (3) judicial costs (corresponding to criminal and civil matters); and (4) prison costs.

The costs of prior counseling and free legal aid include those provided through the permanent and specialized duty roster of the Bar Associations, to which women victims of GBV are entitled. Ultimately, the public sector pays the costs. They were determined based on the XVII Informe del Observatorio de Justicia Gratuita (Observatorio de Justicia Gratuita, 2023).

Police costs were determined based on the amounts allocated by public administrations in Spain to programs related to citizen security, which finance the services provided by the State Security Forces and Corps, regional police forces, and local police forces^5^. The primary source is the State-Level Budget Execution Statistics 2022 (Intervención General de la Administración del Estado, 2023). A ratio was applied to these amounts to approximate the proportional part corresponding to GBV, for the determination of which two criteria were used: the ratio of police personnel (in the calculation of the total costs, their addition to scenario A) and the ratio of reported incidents^6^ (in calculating the total costs, their addition to scenarios B and C).

Judicial costs are incurred when cases of GBV are brought before the judicial jurisdiction, and the authority recognizes the existence of criminal indications that will initiate the corresponding criminal and civil proceedings. First, the number of IPVAW cases in court and their proportion of the total (in both criminal and civil jurisdiction) were determined from the figures for total and IPVAW cases entered for 2022 (Consejo General del Poder Judicial, 2023). Secondly, the unit cost was estimated based on the expenditures allocated in 2022 to cover justice services by central and autonomous public administrations. Expenditure on justice at the state level was obtained from the IGAE (Intervención General de la Administración del Estado, 2023). At the autonomous community level, the most recent data available (as of 2020) from the budget settlements of the Autonomous Communities were used (Consejo General del Poder Judicial, n.d.). The total estimated expenditure on justice for Spain as a whole in 2022 was distributed between the criminal and civil jurisdictions according to the percentage of cases filed in each area out of the total number of cases filed (Consejo General del Poder Judicial, 2023).

Criminal proceedings with convictions for IPVAW and prison sentences entail additional costs arising from the aggressor’s imprisonment in a penitentiary institution. These costs were estimated by approximating the average cost incurred by the public sector per inmate, which was applied to the total number of inmates who have been imprisoned due to gender-based violence. This requires information on the inmate population (Secretaría General de Instituciones Penitenciarias, n.d.) of sex and cause, including those of IPVAW, to calculate the ratio of the inmate population (males) per IPVAW over the total inmate population. The costs borne by public administrations for the provision of prison services are mainly incurred at the state level^7^, within the Ministry of the Interior’s program for Penitentiary Centers and Institutions (Intervención General de la Administración del Estado, 2023).

#### 2.2.3. Labor-Productive Domain

The labor-productive domain estimates the costs generated by IPVAW in the spheres of work and production, including both market (paid) and non-market (domestic, unpaid) activities. These costs occur mainly because of the negative impacts of this violence on the health of the victims and because of the need to attend to the legal processes that derive from it. The methodology enables the determination of the type of agent to which this cost is attributed, including the victim herself, family members and relatives, employers, and the public sector.

To determine the type of impact to be considered, the socio-labor situation of the women served as a starting point, distinguishing whether they engage in any paid or unpaid work. Four types of costs were estimated (1 to 4) based on the cases of women victims of violence who do not perform paid work, and six other kinds of costs were calculated (5 to 10) based on the cases of women victims who do perform paid work.

1. Costs due to increased female inactivity and unemployment

This reflects the costs that IPVAW generates due to the increase in inactivity and unemployment of women who are victims of such violence. The victims bear these costs due to the loss of income from not having access to paid employment and the public sector due to decreased social security contributions. The number of women who do not have access to paid work due to experiencing some IPVAW was estimated from the 2019 Macro-Survey and based on the marginal differential in the unemployment rates between women who are not affected and those who are affected by this violence. The average gross annual salary perceived by women in Spain was imputed for these cases (based on the 2021 Wage Structure Survey of the National Institute of Statistics of Spain). Finally, based on the 2022 Social Security budgets, the proportion of gross salary that corresponds to social security contributions the public sector ceases to collect was determined.

2. Costs due to the difficulties of access to education and training

These costs arise from the loss of dedication to education in the short term due to absences caused by violent episodes. First, the number of women who are victims of some form of IPVAW and participate in training activities was estimated (2019 Macro-survey). The days of absence were attributed to them according to the degree of health impact (Mañas-Alcón et al., 2019). The cost per day was approximated from the outlays involved in annual enrolment in a university degree (Observatorio del Sistema Universitario, 2016) or a higher vocational training degree in the Comunidad de Madrid, Spain, in 2022 (Comunidad de Madrid, 2023). Survivor women bear these costs.

3. Costs due to the difficulties in the provision of domestic services

These are losses caused by the difficulty women face in performing unpaid domestic work due to IPVAW. These were assigned to the victims. Although these activities are non-market activities, it was possible to monetize the economic value they generate based on the hypothesis that in the face of violent episodes, they cannot be provided by women in the same way as in the absence of violence (hours and quality of dedication), or other family members or relatives must deliver them without remuneration or by persons outside the household with remuneration. Once the number of women who perform unpaid domestic work and have experienced some IPVAW was determined, these women were attributed days of absence according to the degree of injuries (2019 Macro-survey). The average gross salary of household employees was considered (Spain Household Employees Wage Tables of 2022), and the average time women dedicate to domestic work, i.e., four hours per day, was based on the Time Use Survey of 2009–2010 by the National Institute of Statistics of Spain.

4. Costs due to difficulties in caring for children

In the same way, it is possible to monetize the losses for the care of children not delivered by women victims of IPVAW. These costs include the losses caused by women victims’ difficulty caring for persons under 18 years of age who live with them at home (2019 Macro-survey). Considerations and sources such as those presented for domestic services costs were applied to this group based on the average gross salary of household employees (Spain Household Employees Wage Tables of 2022) and women’s average time of dedication to care for children: two hours per day, according to the Time Use Survey 2009–2010 of the National Institute of Statistics of Spain.

5. Wage penalty costs for absences due to medical and legal issues.

This category includes the costs incurred due to the loss of work hours resulting from the absenteeism of women victims from their workplaces to receive medical care, attend legal or police procedures, and seek professional help or guidance. These costs are borne by the victims and by the families and relatives who accompany the victims and must also be absent from work or school. Based on the literature (Martínez Martín et al., 2004; Zhang et al., 2012), we considered that female victims spend away from their workplace three days per year. Losses of wage earnings per day were imputed using the average wage for women from the 2021 Wage Structure Survey. In the case of costs borne by the families or relatives of women victims, the same days of absence were attributed, and the wage equivalent to that of a female household worker in 2022 was applied.

6. Costs for sick leave from work due to physical injuries and mental health damages

Here, we estimated the temporary disability benefits to which female worker victims are entitled because of physical injuries and mental health damages due to IPVAW. First, we identified women who have suffered physical injuries as a result of IPVAW and who stated that they have suffered some of the symptoms of mental health damage or have been emotionally affected by this violence (depression; loss of self-esteem; anxiety/phobias/panic attacks; despair; feelings of helplessness; concentration problems, forgetfulness; sleep or eating problems; recurrent pain in some parts of the body; self-injury/thoughts of suicide). We consider that the number of sick leave days attributed to each case varies depending on the severity of injuries or damages (seven and fifteen sick leave days for moderate and severe cases, respectively). These temporary disability costs were distributed between the employer and the public sector in applying labor legislation (depending on the duration of the sick leave).

7. Costs for loss of production for absences due to physical injury and mental health impairment

Absences from work due to physical or psychological damages resulting from IPVAW add another type of cost borne by firms and the public sector since the salary paid to a worker can be considered not only as a cost but also as an investment made by these organizations with a specific expected marginal rate of return per working person. Estimating these costs regarding lost net economic returns arising from IPVAW was based on an expected marginal rate of return on investment for a worker of around 4.8%, indicating that if a company invests an additional 100 euro in a worker, it expects to receive a net return or profit of 4.8 euro (Zhang et al., 2012, p. 66; Souto-Nieves, 2003, p. 19).

8. Costs for reduced productivity due to delays and distractions at work

The physical, sexual, and/or psychological injuries suffered by female victimized workers lead to exhaustion, delays, and work distractions that reduce their productivity, impacting firms. In this respect, Zhang et al. (2012, p. 66) and Reeves and O’Leary-Kelly (2007, p. 39) estimated the percentage of wages accounted for as lost productivity is 12.3% for women who are victims of IPVAW and 8.4% for women who are not victims of IPVAW, which means that the difference in the percentage of wages accounted for as lost productivity due to IPVAW is 3.9%. Thus, to approximate the differential costs arising from work delays and distractions for female employees affected by IPVAW, we applied this differential percentage

9. Costs for employers’ administrative actions due to absences from work

Employers bear these costs for the management tasks they must perform when their female workers are absent from workplaces due to IPVAW (such as reorganizing work, or in case of dismissal or resignation, employers also incur recruitment and training costs of new workers). The estimate of time lost was based on Zhang et al. (2012, p. 67), which considered that managers lose half an hour of productivity per day due to employee absenteeism. The hourly wage of directors and managers was obtained from the 2021 Wage Structure Survey of the National Institute of Statistics of Spain.

10. Cost of benefits paid for job losses

As a consequence of absences, delays, and/or distraction at work, female workers who are victims of IPVAW may end up losing their jobs either because of dismissal or resignation. Cruz Roja Española (2017, p. 53) estimated that 5% of women of working age who are not working are in this situation due to frequent absenteeism from their workplace due to problems with their ex-partner. This percentage was combined with information from the 2019 Macro-survey on women victims of this violence who are not working and who are of working age. The economic costs were estimated based on the incomes perceived by these women, considering the Spanish Public Indicator of Multiple Effect Income (IPREM) in 2022. It is a reference indicator for determining eligibility for various social and economic benefits, grants, and subsidies in Spain.

#### 2.2.4. Other Costs

These include the costs arising from the need for some victims to deal with relocations and removals and to use housing solutions provided by shelters because of the violent episodes suffered as well as the costs faced by organizations to provide various support services to these victims. Alternative scenarios were also defined that consider, on the one hand, women victims of PSV (scenarios A and B) and, on the other hand, women who have suffered PSEVF (scenario C).

The determination of unit costs and the number of cases to which they apply was based on KPMG (2014), which estimates the cost of services delivered by third-sector organizations at 69.5 euros per person, and the 2019 Macro-survey, which enables the identification of the number of victims who have reported contacting shelters or third-sector organizations.

**Figure 4:**
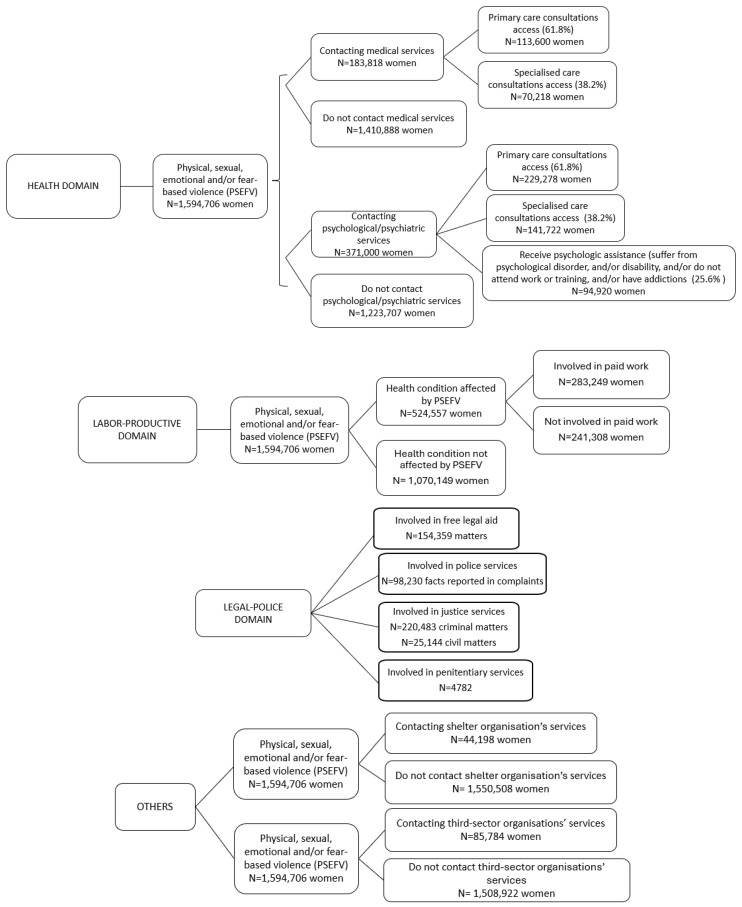
The population of women affected by PSEVF for whom the costs of the different domains are estimated in Scenario C. Source: The authors.

The population of women affected by PSEVF for whom the costs of the different domains are estimated in Scenario C is shown in Figure 4.

## 3. Results

As is shown in Table 1, the total direct tangible costs of IPVAW in Spain in 2022 range from EUR 1.38 billion to EUR 3.01 billion. These figures are equivalent to approximately 0.10% to 0.22% of the GDP and 29 euros to 86 euros per capita.

The costs of the legal-police domain are by far the largest, accounting for a percentage of the total cost that ranges from 37.2% in scenario C to 55.2% in scenario B. Costs in the health domain also have a very significant weight, between 20% in scenario B and 32.2% in scenario C. Labor costs have a more modest weight in scenario A but account for almost a fifth in scenarios B and C. Other costs are the least important item, although they account for just over 11% in scenario C. It should be noted that the health and legal areas, whose costs are borne entirely by the public sector, have a combined weight ranging from 69.4% to 82.9%

The costs associated with women victims of PSEVF (Scenario C) are between 1.5 and 2 times the costs associated with women victims of PSV (Scenarios A and B).

By type of agent (Table 2), following the result of scenario C, the costs of IPVAW are mainly borne by the public sector (85%), followed by women victims (8.2%), employers (5%), family and friends (1.6%), and the third sector (0.2%).

Focusing on the healthcare domain in greater depth, scenario C in Table 3 shows that the costs for care processes are the highest at 76.3%. Within these, the costs arising from specialized care consultations stand out above the rest (61.9%), especially those incurred by hospitalizations (54.4%), followed by primary care services (8.6%), the lowest being the costs of psychotherapy treatments (5.9%).

Policies for the comprehensive prevention of gender-based violence have a relative weighting of 21.8% of total costs. Pharmaceutical costs account for a small percentage.

Paying attention to the costs of the legal-police domain, scenario C in Table 4 shows that police costs are by far the highest, reaching 71.8% of the total. Justice and penitentiary services costs also account for a significant amount, close to 13%. The minor costs come from free legal advice and assistance (2.8%).

Table 5 shows the detailed results in the labor-productive domain in scenario C. The costs attributed to women who are employed and suffer from IPVAW are higher than those attributed to the inactivity or unemployment of women victims. They account for 69.5% and 30.5% of the total costs of this domain, respectively. Within the costs derived from the situation of inactivity or unemployment, the costs originating from the increase in female inactivity are the most significant (22.5%), followed, albeit significantly more distantly, by losses due to difficulties in providing childcare and domestic services resulting from the violence suffered (with 3.9% and 3.6% of the total cost, respectively). Considering the costs from women who are employed, the most significant costs came from the losses incurred by sick leave due to physical injuries and, above all, due to the psychological damage suffered by women victims (these costs together account for 34.7% of the total costs) and from the victims’ loss of earnings for health and legal issues (21.4%).

## 4. Discussion and Conclusions

### 4.1. Results and Public Policy Recommendations

The work developed here demonstrates that having measurements that approximate the costs borne by countries due to IPVAW’s multidimensional impacts is crucial both to ascertain the scope of the losses to society and to raise awareness of its widespread presence and the pervasiveness of its effects.

In addition, a better understanding of its impacts and the distribution of its costs can contribute to the more effective design of public policies for prevention and eradication.

The microeconomic approach to applying accounting methodology enables the approximation and monetization of the tangible direct costs of this violence in Spain in a comprehensive and detailed manner both in terms of the types of costs and the agents that bear them. The results obtained for Spain, as summarized in Section 3, show that these forms of violence have a profound economic impact on society as a whole, reaching a cost of 3015 million euros per year or 0.22% of GDP.

These results are in line with the findings from other studies for numerous countries, including advanced economies: 0.14% in France (Nectoux et al., 2010); 0.47% in Canada (Zhang et al., 2012); 0.35% in Switzerland (Stern et al., 2013); and 0.8% in the European Union (Walby & Olive, 2014)^8^. The results obtained in this work are conservative if they are compared with their results obtained in the study of economic costs of gender-based violence in the EU developed by EIGE (EIGE, 2021); it estimated 15,975 million euros as the cost of intimate partner violence against women in Spain, equivalent to 1.27% of GDP.

In addition, the distribution by affected agents of the estimated costs for Spain, with 85% being borne by the public sector and 5% by employers, provides clear evidence that the impact of these forms of IPVAW extends beyond the sphere of the intimate relationships where it originates and reaches other spheres in which various economic and social agents are also involved and affected. The work shows this form of violence against women also more significantly affects the legal (37% of total cost) and health (32%) services systems dependent on the different levels of public administrations. Additionally, the costs borne by private and public employers and work environments, including domestic non-paid work (19%) as well as other types of costs incurred by private social services dependent on third-sector associations (11%), are relevant.

The three modeling scenarios considered not only allow us to obtain a range of measures of the tangible direct costs borne by Spain in a year because of IPVAW but also provide a highly detailed understanding of the mechanisms through which this violence impacts the four domains defined in this study: healthcare, legal-police, labor-productive, and other costs. It also provides information on the actors who bear these costs, including women victims, families and relatives, the public sector, employers, and third-sector organizations.

At the same time, given that three possible scenarios are based on different groups of women victims according to the type of gender-based violence suffered (physical and/or sexual violence with limiting injuries (scenario A), physical and/or sexual violence according to the severity of the violence (scenario B), and physical, sexual, emotional, and/or fear-based violence (scenario C)), the results of the estimations offer a broad vision of the phenomenon of gender-based violence conditioned by the type of group used in each of these scenarios. This strategy aligns with EIGE’s recommendation to use a range of values or different data sources to acknowledge uncertainties in the extent of gender-based violence within a country (EIGE, 2021, p. 47).

In addition, the various scenarios allow us to formulate public policy recommendations on a specific collective of survivors of this violence. In other words, it is possible to visualize the economic cost of more invisible forms of intimate partner violence, such as emotional violence and fear (PSEVF). These different scenarios enable the development of sensitivity analysis, considering not only various groups of survivors but also various values of key parameters used in the models. For example, the average wages and salaries of women workers, the rates of labor absenteeism, the rate of over-frequentation of healthcare services by survivors, the prices of medicines, and the cost of healthcare treatments, among others related to labor market characteristics, can be mentioned.

The estimates presented are based on microdata from the 2019 Macro-survey, designed explicitly for the study of violence against women in Spain, with a high level of detail and statistical quality. The abundant information from this source was combined with an extensive set of other sources of information, primarily official, with a common element in almost all of them: they all refer to the Spanish reality. In contrast, other studies have extrapolated unit costs from different countries as well as prevalence rates. For example, the referenced study from EIGE estimated the costs of intimate partner violence against women for the EU based on the extrapolation of the estimate made for the United Kingdom, weighting them according to population criteria (EIGE, 2021).

Most cost studies provide estimates for IPVAW of a physical and/or sexual nature, which discounts the impacts of psychological violence, which is the most common type of violence. The estimates proposed here, in scenario C, do take into consideration these forms of violence by incorporating emotional violence and cases in which the woman discloses that she is or has been afraid of her current or past partner. In this way, we offer the closest results to reality in the broadest scenario (C).

The distribution of tangible costs of IPVAW in Spain by domain is not easily comparable with other studies due to differences in concepts of violence, types of costs, and methodologies. However, it is worth noting one of the most recent estimations for the United Kingdom (EIGE, 2021), based on the work of Heeks et al. (2018). Considering tangible costs only, the highest costs from IPVAW also came from the criminal justice system, including police costs, which accounted for more than 40%. However, the economic costs of health services are lower, while they are higher for the lost economic output compared with Spain.

### 4.2. Limitations and Future Research Lines

Although the estimates presented here encompass a broad range of IPVAW impacts and costs, they are not without limitations.

First, one drawback attributed to the accounting method is the tendency to double-count costs; i.e., the exact costs can be accounted for under different domains or by various agents, who in many cases cannot even be correctly identified. In this work, a rigorous analysis was conducted to prevent double-counting of costs, providing estimates with sufficient guarantees based on high-quality information sources. In contrast, the monetized cost of IPVAW obtained for the Spain case most likely underestimates the real losses of this phenomenon for various reasons.

On the one hand, due to difficulties in information disposal and others, some crucial costs can be overlooked, such as emotional costs that extend far beyond the monetary.

Thus, not all tangible direct costs were measured, generally due to information difficulties (e.g., those generated in the workplace as a result of absences, delays, and distractions of the perpetrators or those resulting from health damage to the children of women who are victims of violence).

It should be noted that the direct intangible costs, derived from the pain and suffering of the survivors and their children and from the loss of life (of the women victims, their children, and the aggressors themselves), were not included.

The second-round costs (indirect and induced) were not calculated either, and the microeconomic perspective used to estimate the tangible direct costs was not complemented by adopting a macroeconomic perspective. Thus, such an approach would enable the assessment of the demand impacts resulting from direct costs on the economy as a whole, through the economic multipliers that amplify the initial direct impact. Evidence from other studies suggests that the costs associated with considering both categories would be very high.

It is worth noting that another limitation of the study is its cross-sectional approach. The estimates were based on prevalence-year figures. This consideration, although necessary from an operational point of view, is a restriction because it excludes from the estimates those women victims who have suffered violence before the twelve months before the interviews and who continue to show symptoms of physical or mental health injuries and therefore need to utilize health services or follow medical treatment. Also, we did not consider those who continue to be immersed in legal or judicial proceedings or who, as a consequence of the ailments that limit their daily activity, continue to be on temporary sick leave or have to be absent from their place of work or studies to attend to legal or judicial proceedings related to the violent episodes, considering that all of these events occurred not only before twelve months before the interviews but also much before.

Therefore, this situation results in current disbursements from the victims themselves, their families and relatives, and above all from the public sector because of past violence, which is not taken into account in the monetization of costs.

Further research is needed to continue exploring the impacts and economic costs of IPVAW as well as the complementarity between different cost methodologies to reveal the micro- and macroeconomic losses associated with this form of violence.

## Figures and Tables

**Table 1:** Summary of estimates of the direct tangible costs of IPVAW in Spain in 2022. Details by domain. Absolute values (EUR million), distribution (%), and percentage of GDP in 2022. Source: The authors, Spanish National Accounts (INE), and Population Census on 1 January 2022 (INE).

	IPVAW Costs: Three Modeling Scenarios					
	A		B		C	
	EUR Million	%	EUR Million	%	EUR Million	%
Healthcare	409.91	29.7	409.91	20.2	969.61	32.2
Legal-police	733.10	53.2	1121.80	55.2	1121.80	37.2
Labor-productive	156.85	11.4	423.71	20.8	585.14	19.4
Other costs	78.41	5.7	78.41	3.9	338.05	11.2
Total Tangible Costs	1378.27	100	2033.83	100	3014.61	100
Total tangible costs (% of GDP)	0.10		0.15		0.22	
Tangible costs per person (EUR)	29		43		64	
GDP and population data for Spain						
GDP of Spain at current prices 2022 (EUR million)	1,373,627 *					
Population of Spain (people)	47,432,805					

* Figure checked on 23 March 2025.

**Table 2:** Summary of scenario C estimates of the direct tangible costs of IPVAW in Spain in 2022. Details by domain and agent. Amounts in euros. Source: The authors.

	Survivor Women	Families and Relatives	Employers	Public Sector	Third Sector-Party
Healthcare				969,609,866	
Legal-police				1,121,802,198	
Labor-productive	216,807,479	49,177,350	149,514,445	169,645,360	
Other costs	30,629,214			300,420,794	6,999,374
Total Tangible Costs	247,436,693	49,177,350	149,514,445	2,561,478,218	6,999,374
% of total	8.2	1.6	5.0	85.0	0.2

**Table 3:** The Costs of IPVAW in the Healthcare Domain. Scenario C.

	Amounts (EUR)	% of Total
A. Care Processes per Patient	739,661,522	76.3
A.1. Primary Care Consultations	82,963,520	8.6
- Doctors	72,937,005	7.5
- Nursing	10,026,516	1.0
A.2. Specialized Care Consultations	599,720,194	61.9
- Outpatient consultations	30,480,096	3.1
- Emergencies	34,168,630	3.5
- Hospitalization	527,650,227	54.4
- Mobile transfer	7,421,241	0.8
A.3. Treatments Psychotherapies	56,977,807	5.9
B. Pharmaceutical Costs	18,716,015	1.9
C. Prevention policies	211,232,330	21.8
Total Healthcare Domain	969,609,866	100.0

Source: The authors.

**Table 4:** The Costs of IPVAW in the Legal-Police Domain. Scenario C.

	Amounts (EUR)	% of Total
Legal advice and aid	31,013,818	2.8
Police services	804,919,386	71.8
Justice services	146,922,792	13.1
Criminal matters	132,169,256	11.8
Civil matters	14,753,536	1.3
Penitentiary institutions	138,946,202	12.4
Total Legal-Police Domain	1,121,802,198	100.0

Source: The authors.

**Table 5:** The Cost of IPVAW in the Labor-productive Domain. Scenario C.

	Amounts (EUR)	% of Total
Women in Inactivity and Unemployment		
Increase in females dropping out of the workforce and unemployment	131,376,557	22.5
Difficulty in education and training	3,162,161	0.5
Difficulty in unpaid domestic work	21,211,597	3.6
Difficulty caring for children	23,007,647	3.9
Women in Employment		
Loss of wages for absences due to medical care and legal matters	125,065,685	21.4
Sick leave due to injury	11,367,220	1.9
Mental health sick leave	191,514,898	32.7
Production losses due to absenteeism	9,738,342	1.7
Decrease in productivity	43,676,541	7.5
Official bodies’ formalities for employers	17,575,121	3.0
Benefits for loss of employment	7,448,865	1.3
Total Labor-Productive Domain	585,144,634	100

Source: The authors.

## Data Availability

Data are contained within the article.

## Notes

1 In Spain, the first and most important legal norm in this topic was developed in 2004: Ley Orgánica 1/2004, de 28 de diciembre, de Medidas de Protección Integral contra la Violencia de Género (Agencia Estatal Boletín Oficial del Estado, 2024). This law refers to IPVAW as gender-based violence.

2 For the costs of the legal-police domain, the prevalence of administrative records is considered, which refers to the cases reported and detected by official institutions, generating a demand for services in this area. Institutional prevalences yield much lower figures than the prevalences derived from self-perception surveys, given the low percentage of reports of these forms of violence.

3 Its first two modules focus on the study of violence against women by current (module 1) or former (module 2) partners.

4 Emotional violence is a specific type of psychological violence that includes the following behaviours by a partner or former partner: he has insulted you or made you feel bad about yourself; put you down or humiliated you in front of other people; frightened or intimidated you on purpose; verbally threatened to hurt you; verbally threatened to hurt your children or someone else who is important to you; threatened to harm himself if you leave him; threatened to take your children away from you (according to the 2019 Macro-survey).

5 The Expenditure Programs of the responsible bodies at the state (Ministry of the Interior), regional (for the three Autonomous Communities that have their police forces), and local levels have been reviewed. The amounts of recognized obligations or executed expenditures have been considered whenever possible, and only those corresponding to wages and salaries as well as expenditures on goods and services necessary for the provision of services have been considered.

6 These ratios are derived from Secretaría de Estado de Presupuestos y Gastos (2023) and Ministerio del Interior (2022).

7 Catalonia and the Basque Country have also transferred these competencies.

8 These comparisons should be taken with due caution, as methodologies and concepts are not homogeneous or fully comparable in a strict sense.

## Abbreviations

The following abbreviations are used in this manuscript:

IPVAW	Intimate partner violence against women
GVB	Gender-based violence
PSV	Physical and/or sexual IPVAW
PSEVF	Physical, sexual, and/or emotional IPVAW and/or fear
WHO	World Health Organization
DGVG	Delegación del Gobierno contra la Violencia de Género (Government Office against Gender-Based Violence)
INE	Instituto Nacional de Estadística (The Spanish National Institute of Statistics)
EIGE	European Institute for Gender Equality
ICRW	International Center for Research on Women
MSSSI	Ministerio de Sanidad, Servicios Sociales, e Igualdad (Ministry of Health, Social Services and Equality)
NCIP&C	National Center for Injury Prevention and Control (USA)
NCRVAW&C	National Council to Reduce Violence against Women and their Children (Australia)
UNFPA	United Nations Fund Population
